# Determinants of care-seeking for ARI/Pneumonia-like symptoms among under-2 children in urban slums in and around Dhaka City, Bangladesh

**DOI:** 10.1038/s41598-024-80979-x

**Published:** 2025-03-29

**Authors:** Samiun Nazrin Bente Kamal Tune, Gulam Muhammed Al Kibria, Mohammad Zahirul Islam, Md. Arif Billah, Maya Vandenent, Md. Shamim Hayder Talukder, Ummay Ferhin Sultana, Maliha Khan Majlish, Shafiun Nahin Shimul, Margub Aref Jahangir, Jahangir A.M. Khan, Shahin Akter, Kazi Fayzus Salahin, Md. Razib Chowdhury, Abdur Razzaque, Taufique Joarder

**Affiliations:** 1 International Development Division (IDD), Abt Global, Dhaka, 1212 Bangladesh; 2 Johns Hopkins University Bloomberg School of Public Health, Baltimore, MD 21205 USA; 3 Development Cooperation Section, Embassy of Sweden, Dhaka, Bangladesh; 4 Health Systems and Population Studies Division, icddr,b, 68 Shaheed Tajuddin Ahmed Sarani, Mohakhali, 1212 Dhaka Bangladesh; 5 UNICEF, Dhaka, Bangladesh; 6 Eminence Associates for Social Development, Dhaka, Bangladesh; 7 Institute of Health Economics, University of Dhaka, Ramna, 1000 Dhaka Bangladesh; 8 Health Economics and Policy Unit, School of Public Health and Community Medicine, Institute of Medicine, University of Gothenburg, Gothenburg, Sweden; 9 SingHealth Duke-NUS Global Health Institute, Singapore, Singapore

**Keywords:** Public health, Risk factors

## Abstract

Childhood pneumonia affects an estimated 18% of under-five children in Bangladesh. Urban slum-dwellers face challenges in healthcare-seeking. This study examined the factors influencing the healthcare-seeking for childhood pneumonia among under-two children in urban slums in Bangladesh. The study examined influence of children’s characteristics (age, sex, number of ARI/pneumonia symptoms, and duration of symptoms), maternal factors (age, education, and working status), and household characteristics (number of household members, wealth quintile, sex of household heads, age of household heads). The outcome variable was receiving care from a qualified medical provider for childhood pneumonia or pneumonia-like symptoms within 14 days before the collection of surveillance data. The research utilized data from the Urban Health and Demographic Surveillance System, which included 155,000 people from five slums in Dhaka and Gazipur City Corporation areas. Overall, 753 out of 4,679 (16%) children under two years of age were included in this study, all of whom had ARI/pneumonia-like symptoms. The mean age of these children was 11.4 months, and 50% were boys. Of them, 350 (46%) sought care from local pharmacies, while 37% sought care from medically trained providers. Logistic regression analyses indicated that children with multiple symptoms (AOR: 2.32, 95% CI: 1.71–3.14) and illness duration over seven days (AOR: 2.61, 95% CI: 1.51–4.51) had higher odds of receiving care from a medically trained provider. Higher maternal education compared to no formal education, having five or more household members compared to four or fewer, household heads aged 40–49 years compared to those under 25 years, a longer duration of living in the slum (more than 10 years compared to less than five years), and belonging to the richest wealth quintile compared to the poorest were protective factors for care-seeking from qualified providers. Further research is required to understand the context for designing appropriate interventions and comprehensive policies for improved child health regarding ARI/pneumonia-like symptoms.

## Introduction

Childhood pneumonia, a form of acute respiratory infection (ARI), is a global public health concern^1^. Children under five years of age are still vulnerable to this condition worldwide, according to the World Health Organization^2^. Globally, an estimated one out of every 71 children is diagnosed with childhood pneumonia each year^3^. Although Bangladesh is one of the lower-middle-income countries (LMICs) in South Asia that achieved the Millennium Development Goal 4 by reducing child mortality^4^, pneumonia remains a major cause of death among under-five children in this country, responsible for 18% of under-five deaths^5^. Preventive and promotive interventions, including pneumococcal conjugate vaccine (PCV), Hemophilus influenza type B (Hib) vaccination, oral antibiotics, pulse oximetry, and oxygen, have been strengthened to prevent pneumonia-related deaths among under-five children in Bangladesh^6,7^. Limited resources for medications and hospital-based management create crucial challenges in the LMICs^6^, hindering progress and obstructing indicators of Sustainable Development Goal 3 (SDG 3) (Target 3.8.1), which emphasizes the importance of seeking care for children with ARIs^8^. Although progress has been made, the rate of seeking care for children with ARIs stands at 46.4% in Bangladesh^9^.

Global Action Plan for Pneumonia and Diarrhea (GAPPD), a collaborative initiative by the World Health Organization (WHO) and the United Nations Children’s Fund (UNICEF), aimed to improve access to healthcare and treatment for childhood pneumonia^10^. The quality of care by health professionals depends on evidence-based approaches for better health outcomes^11^. The concept of ‘Any Qualified Provider’ (AQP) refers to the healthcare practitioners fulfilling the required criteria established by the National Health Service (NHS), including education, training, certification, license, ethical adherence, continuous professional growth, and accreditation by regulatory bodies^12^. Health workers are classified by the International Standard Classification of Occupations (ISCO) into five groups, with health professionals at the forefront, generally engaged in preventive, curative, and rehabilitative health services^13^. The Bangladesh Ministry of Health and Family Welfare (MOHFW) utilized the ISCO-2008 Classification for the Human Resources for Health (HRH) Country Profile, with support from the WHO^14^. This classification covers various healthcare providers, including medical practitioners, medical assistants, dentists, pharmacy professionals (with bachelor and diploma-level education), nursing professionals, midwives, and medical technologists, along with community health workers and practitioners of Ayurveda, Unani, and homeopathy medicine^15,16^. About 77% of the recognized and qualified health workers, according to ISCO-08/ Bangladesh Standard Classification of Occupations (BSCO) 2012 version, are situated in urban Bangladesh^13^.

A large disparity in pneumonia-related healthcare-seeking behavior among guardians of children was observed between the richest and the poorest wealth quintiles in Bangladesh^17^. There is a fundamental gap in preparedness concerning pneumonia management based on the Integrated Management of Childhood Illness (IMCI)within the public health infrastructure of Bangladesh^18^. More than 90% of district hospitals and upazila health complexes, alongside certain lower-level facilities, provide IMCI-based pneumonia management services, but unfortunately, less than two-thirds of the staff members have received requisite training in IMCI-based pneumonia treatment protocols^19^.

Approximately 38% (62.5 million) of the total population in Bangladesh resides in urban areas, and around half of these urban people live in slums^19^. Within the urban settings, most children diagnosed with pneumonia from the wealthiest quintile receive medical care from either private or public healthcare facilities^17,20^. On the other hand, patients belonging to the most economically challenged quintile seek treatment for pneumonia from pharmacies or drug stores and, occasionally, from traditional healers^17,20^. Residents of urban slums predominantly choose local pharmacies for the treatment of different illnesses due to their economic viability, accessibility, and proximity^21^. Periodically, care is sought from informal healthcare providers, such as traditional healers or *kabiraj* as there is a visible avoidance of public facilities stemming from perceived negligence of the authority or dissatisfaction over the quality of services provided^21,22^. The Bangladesh Demographic and Health Survey (BDHS) reported that the under-five mortality rate was 46 per 1000 livebirths in 2014^23^ at the national level, and the rate among residents in urban slums was 57 per 1000 livebirths in 2012^23^.

Urban slum residents face severe challenges regarding access to healthcare due to inadequate facilities, poor living conditions, high air pollution, forced migration, and extreme population density^24,25^. Therefore, it is essential to comprehend the healthcare-seeking behavior of this segment of the population. However, to our current knowledge, there is a dearth of data on care-seeking behavior for childhood pneumonia among urban slum-dwellers in Bangladesh. This paper, thus, aimed to identify the factors associated with seeking care for childhood pneumonia from a qualified medical practitioner among urban slum-dwellers in Bangladesh. The findings of our study will be helpful for researchers, program managers, and policymakers to design effective programs and policies to bring this vulnerable pediatric population group under treatment coverage by qualified providers.

## Conceptual framework

**Fig. 1:**
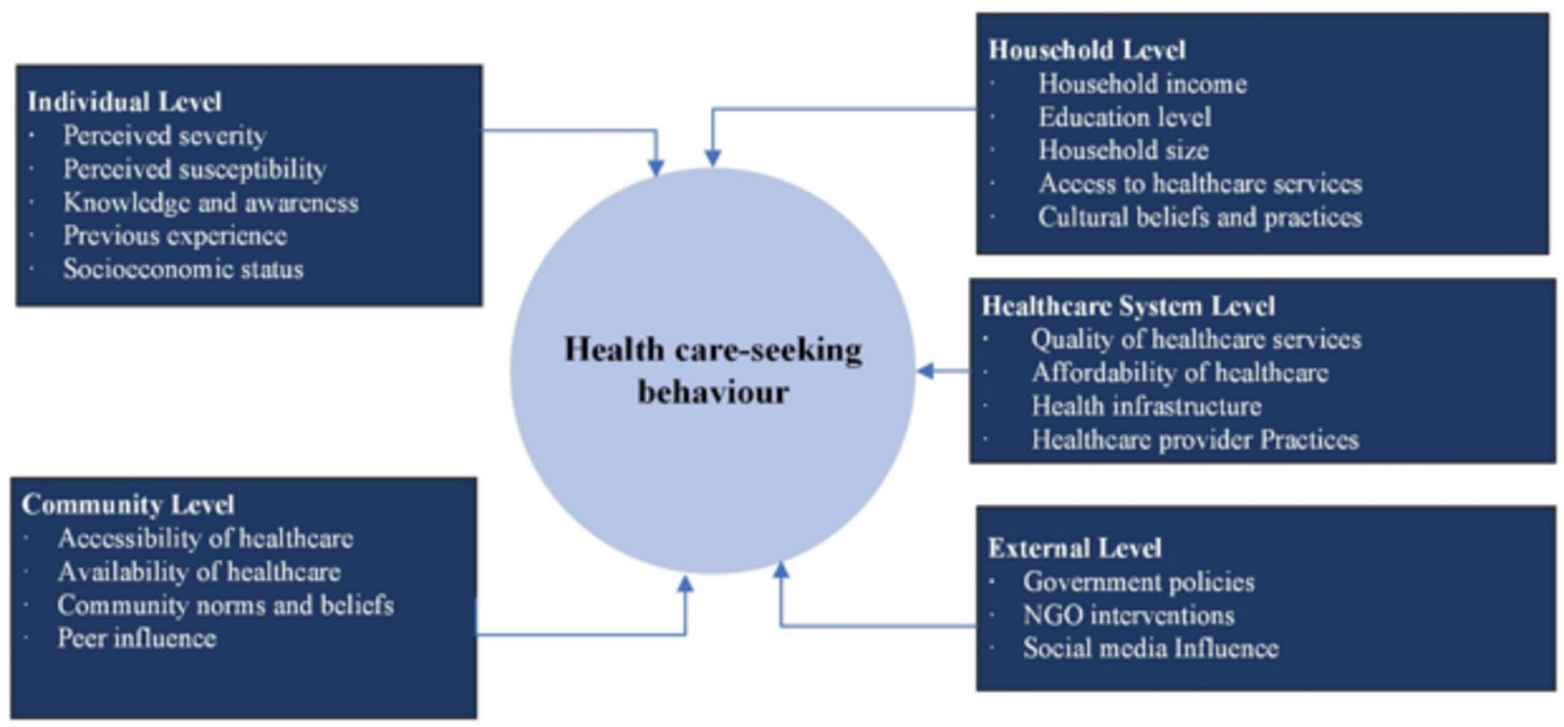
Conceptual framework of the study. Adapted from literature review^17,21,22,25–28^

The conceptual framework of this study (Fig. 1), developed from a literature review, illustrates various determinants operating at the individual, household, community, healthcare services, and external levels or some other contextual factors, such as government policies, interventions by non-governmental organizations (NGOs), and social influences^17,21,22,24–27^. These factors influence the decision-making process regarding seeking care for ARI or pneumonia symptoms among under-five children residing in urban slums of Bangladesh, with their interactions intricately shaping care-seeking behavior.

## Materials and methods

### Study design and settings

The data of this study were extracted from the Urban Health and Demographic Surveillance System (Urban HDSS), a community-level prospective cohort study of 155,000 population of five selected slums of Dhaka (North and South City Corporations) and Gazipur City Corporations. The Urban HDSS has been being maintained by icddr, b (an international health research institution located in Dhaka, Bangladesh) since 2016. The study area is in proximity to where people of middle- and high-income groups live, and many garment factories are there.

The people living in urban slums are at risk of contracting diseases as the environment in the slum is favorable for transmission due to the overcrowded living conditions and limited access to public health infrastructure^28,29^. In these slums, most households possess one bedroom with a mean dwelling area of 119 square feet. Most households used pipe water for drinking, while only one-third had access to sanitary latrines that flush to sewerage/septic tanks. Sharing of water sources, latrines, and cooking places was very common in the slums^30^.

### Study participants

**Fig. 2:**
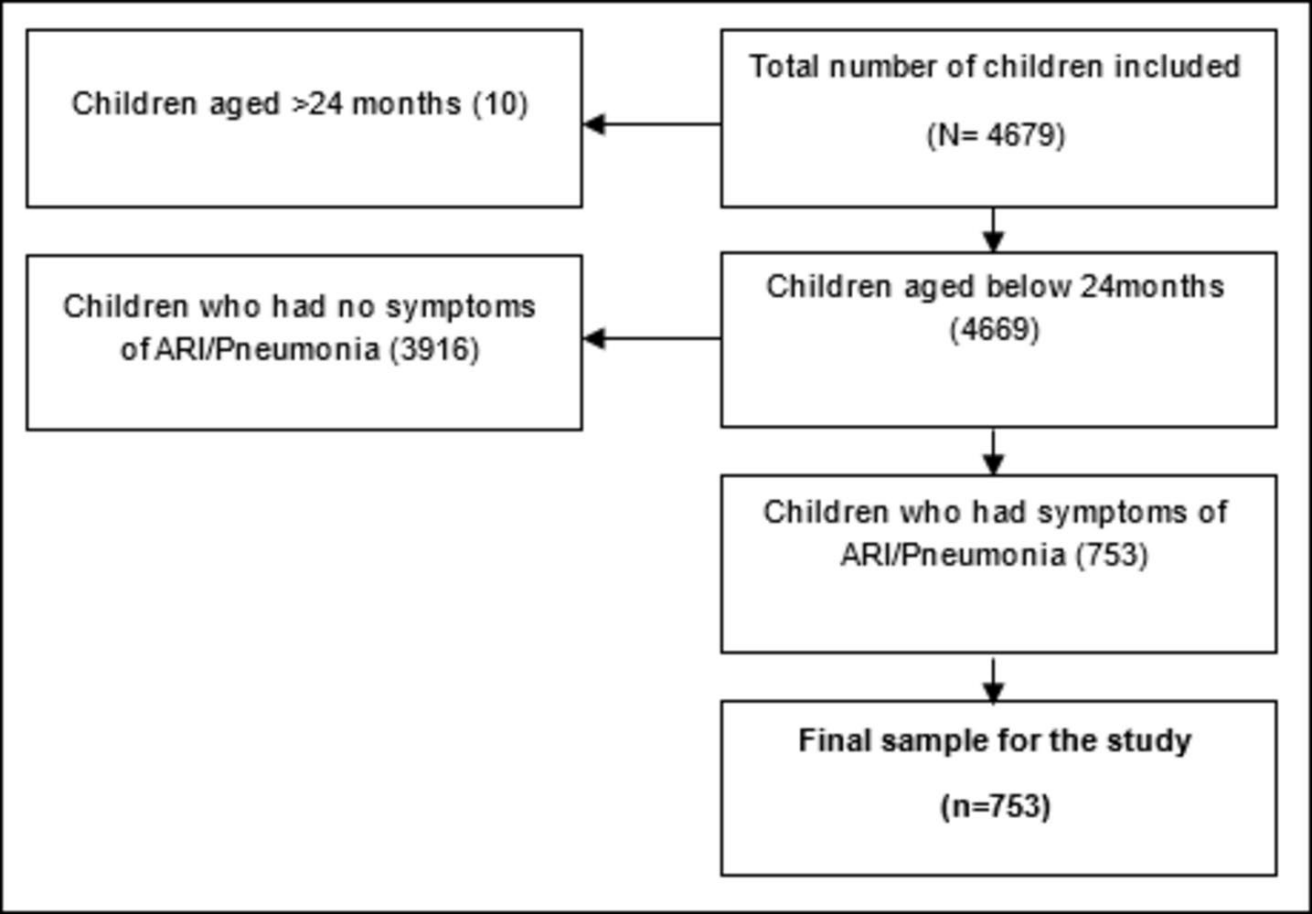
Selection of the sample.

In this study, we used data from a single round of Urban HDSS, collected in 2023 (mid-January to mid-April). The data on childhood ARI/pneumonia-like symptoms were collected via interviews with the mothers^31^. The inclusion criteria of the sample were as follows: (i) Children should be aged below 24 months, (ii) Must live in the Urban HDSS sites, and (iii) Reported to have ARI/pneumonia-like symptoms, such as fever, cough, breathing difficulties, and chest in-drawing. Overall, 4,679 children aged under two were included; among them, 753 were reported as having symptoms of ARI/pneumonia. All 753 children with these symptoms were included in this study. The procedure for the selection of the samples is included in Fig. 2.

## Variables of interest

### Associated factors/exposure variables

We extracted a list of associated factors from a comprehensive literature review in several public health databases: PubMed, Scopus, Web of Science, Science Direct, and WHO Repository. Extracted variables were later matched with the existing databases of Urban HDSS, and those exposure variables in the Urban HDSS were selected. Comprehensive literature searches assisted in the categorizations, along with identifying the best possible associated factors for the study. All the associated variables were categorized into three characteristics (children’s, maternal, and household characteristics). These factors are listed below-.

#### Children’s characteristics

- Age.
- Sex.
- Number of ARI/pneumonia symptoms.
- Duration of symptoms.

#### Maternal factors

- Age.
- Education.
- Working status.

#### Household characteristics

- Number of household members.
- Wealth quintile.
- Sex of household heads.
- Age of household heads.
- Duration of living in slums.

Mothers who were in some professions that were considered economically earning for their family were considered ‘working mothers’ who were mostly employed in the private sector as garment workers; others were government workers, NGO workers, self-employed in businesses of their own, or worked as skilled/unskilled laborers, domestic workers, and others. Students, housewives, and physically challenged women who were unable to engage in income-generating activities were not considered working mothers. For the household asset quintiles, the Urban HDSS used a predefined set of household asset lists (including 17 items) possessed by the households or owned by the household members (chair, dining table, *khat*,* chowki* (couch), almirah, sofa set, radio, television, refrigerator, mobile phone, fan, watch, rickshaw, computer, sewing machine, bicycle, and motorcycle). A score was generated from the asset lists for each of the households, using the principal component analyses, and converted into percentiles following ascending order. These percentiles were equally distributed into five categories (1st or lowest to the 5th or highest wealth quintile)^32^.

### Outcome variable

The outcome variable in this study was receiving care from a qualified medical service provider for childhood pneumonia or pneumonia-like symptoms in the last 14 days preceding the interview. Those who reported having any symptoms of ARI/pneumonia-like symptoms, such as fever, cough, breathing difficulties/short breathing, and chest in-drawing for their children, were further asked from where they sought treatment for such symptoms. Those who reported seeking care from any formal private doctors; union-level health facilities, such as Family Welfare Center (FWC), rural dispensaries (RD), hospitals (government/icddr, b, and NGO health facilities, were considered to have sought care from qualified medical service providers. Although FWCs and RDs are primarily rural-based health facilities, our study considered these for internal migration and mobility of the slum population. People living in slum areas often visit their village homes for various reasons and may seek care at nearby rural facilities where they have family members.

### Data collection and quality assurance

Urban HDSS has been conducting data collection quarterly after the baseline census since the end of 2015. The standard protocol was followed to collect data with the help of 25 trained female field workers under the supervision of three field supervisors and a data monitoring team. The data were validated by the data monitoring team. Any inconsistencies reported to the field supervisors were checked by the field workers who consulted available records and conducted the field visits if needed. Field supervisors also observed 2–3% of the households to ensure the quality of data. To minimize reporting errors, female field workers were adequately trained to collect the data, particularly for the date of events and the care-seeking facilities. The training process included the following components:

- Initial Training: Each field worker underwent a 3-day training upon joining, covering the modules and procedures of the training program.
- Practical Experience: Field workers gained hands-on experience by working with more experienced staff members.
- Daily Supervisory Feedback: Continuous feedback was given daily to address any issues and improve accuracy.
- Quarterly Refresher Training: Field workers also participated in quarterly refresher courses to maintain data quality.

### Statistical analysis

First, the participants were described by their background characteristics. Continuous variables (e.g. maternal age) were reported with mean and standard deviation (SD), and categorical variables (e.g. child’s sex) were reported with numbers and percentages (%). The distributions of continuous and categorical variables were compared using Student’s *T*-test and chi-square tests, respectively. Next, the incidence of care-seeking, along with a 95% confidence interval (CI), was reported by the type of medical service provider. Lastly, the unadjusted regression analysis was conducted to examine the association of study variables with the outcomes. Study variables that were significantly associated with the outcomes (*p* < 0.05) in unadjusted analyses were included in a multivariable model to assess their adjusted associations. Both unadjusted and adjusted odds ratios (OR) were reported with a 95% CI. The analysis was conducted by Stata version 15.1 (Stata Corp., College Station, TX, USA).

### Ethical considerations

The whole Urban HDSS study was approved by the Research and Ethical Review Committee of icddr, b (protocol number 15045). Moreover, before the interview, the field workers explained the objectives of the surveillance system and guaranteed the anonymity and confidentiality of the information they would collect from participants. For ARI/pneumonia cases, mothers were interviewed. Besides, informed written consent was obtained from the household head either through a signature or a thumb impression^31^. Thus, all the methods employed in our study were performed in strict accordance with the relevant guidelines and regulations.

## Results

### Characteristics of the study sample

Nearly half (48.34%) of the children in the study were aged between 12 and 23 months, with the majority experiencing illness for a duration of three to seven days (Table 1). More than half of the mothers (53.92%) had attained secondary or higher education; yet, most (87.38%) of them were not employed. The typical household comprised five or more members and was predominantly headed by males (88%). Care from a qualified health service provider was mostly sought for children under six months of age, exhibiting multiple symptoms, and those with illnesses extending beyond seven days. There were significant associations between both maternal education and household wealth concerning the odds of seeking care from a qualified health service provider.

**Table 1:** Characteristics of the study sample (*N* = 753).

Variable	Overall number (%)	Care by the qualified provider		
		Yes (278) *n* (%)	No (475) *n* (%)	*p*-value
Age of the child				< 0.001
Mean (SD)	11.42 (6.29)	9.68 (6.01)	12.44 (6.23)	< 0.001
<6 months	166 (22.05)	83 (50.0)	83 (50.0)	
6–11 months	223 (29.61)	88 (39.46)	135 (60.54)	
12–23 months	364 (48.34)	107 (29.40)	257 (70.60)	
Sex of the child				0.827
Male	378 (50.20)	141 (37.30)	237 (62.70)	
Female	375 (49.80)	137 (36.53)	238 (63.47)	
Number of symptoms				< 0.001
Single symptom	383 (50.86)	105 (27.42)	278 (72.58)	
Multiple symptoms	370 (49.14)	173 (46.76)	197 (53.24)	
Duration of illness				< 0.001
Mean (SD)	6.53 (3.43)	7.12 (3.33)	6.18 (3.45)	< 0.001
<3 days	87 (11.55)	21 (24.14)	66 (75.86)	
3–7 days	391 (51.93)	132 (33.76)	259 (66.24)	
Above 7 days	275 (36.52)	125 (45.45)	150 (54.55)	
Maternal age				0.182
Mean (SD)				0.099
<25 years	25.62 (5.66)	25.91 (5.52)	25.45 (5.75)	0.277
25–34 years	355 (47.14)	118 (42.45)	237 (49.89)	
34 and above	349 (46.35)	143 (51.44)	206 (43.37)	
Maternal education				< 0.01
Mean (SD)	6.12 (3.32)	6.47 (3.04)	5.91 (3.47)	0.027
No formal education	87 (11.55)	20 (7.19)	67 (77.01)	
Up to primary level	260 (34.53)	93 (35.77)	167 (64.23)	
Secondary or higher level	406 (53.92)	165 (40.64)	241 (59.36)	
Mothers’ working status				< 0.05
Not working	658 (87.38)	252 (38.30)	406 (61.70)	
Working	95 (12.62)	26 (27.37)	69 (72.63)	
Number of household members				< 0.01
Mean (SD)	5.80 (2.67)	6.16 (2.69)	5.60 (2.64)	< 0.01
Up to 4	301 (39.97)	92 (30.56)	209 (69.44)	
5 or more	452 (60.03)	186 (41.15)	266 (58.85)	
Household wealth quintile				0.001
First (lowest)	114 (15.14)	21 (18.42)	93 (81.58)	
Second (lower)	135 (17.93)	40 (29.63)	95 (70.37)	
Third (middle)	111 (14.74)	44 (39.64)	67 (60.36)	
Fourth (higher)	226 (30.01)	91 (40.27)	135 (59.73)	
Fifth (highest)	167 (22.18)	82 (49.10)	85 (50.90)	
Sex of the household head				0.308
Male	662 (87.92)	240 (36.25)	422 (63.75)	
Female	91 (12.08)	38 (41.76)	53 (58.24)	
Age of the household head				0.50
Mean (SD)	42.06(14.85)	43.83 (15.20)	41.02 (14.56)	0.012
<30 years	160 (21.25)	46 (28.75)	114 (71.25)	
30–39 years	245 (32.54)	89 (36.33)	156 (63.67)	
40–49 years	133 (17.66)	58 (43.61)	75 (56.39)	
50 and above	215 (28.55)	85 (39.53)	130 (60.47)	
Duration of living in the slum				< 0.01
Mean (SD)	18.20 (22.23)	21.39 (22.57)	16.33 (21.83)	< 0.001
<5 years	355 (47.14)	108 (38.85)	247 (52.00)	
5–9 years	75 (9.96)	33 (11.87)	42 (8.84)	
10 or more years	323 (42.90)	137 (49.28)	186 (39.16)	

The distributions of continuous variables were compared using Student’s t-test, and categorical variables were compared using chi-square tests.

### Management and care-seeking for ARI/Pneumonia-like symptoms

**Fig. 3:**
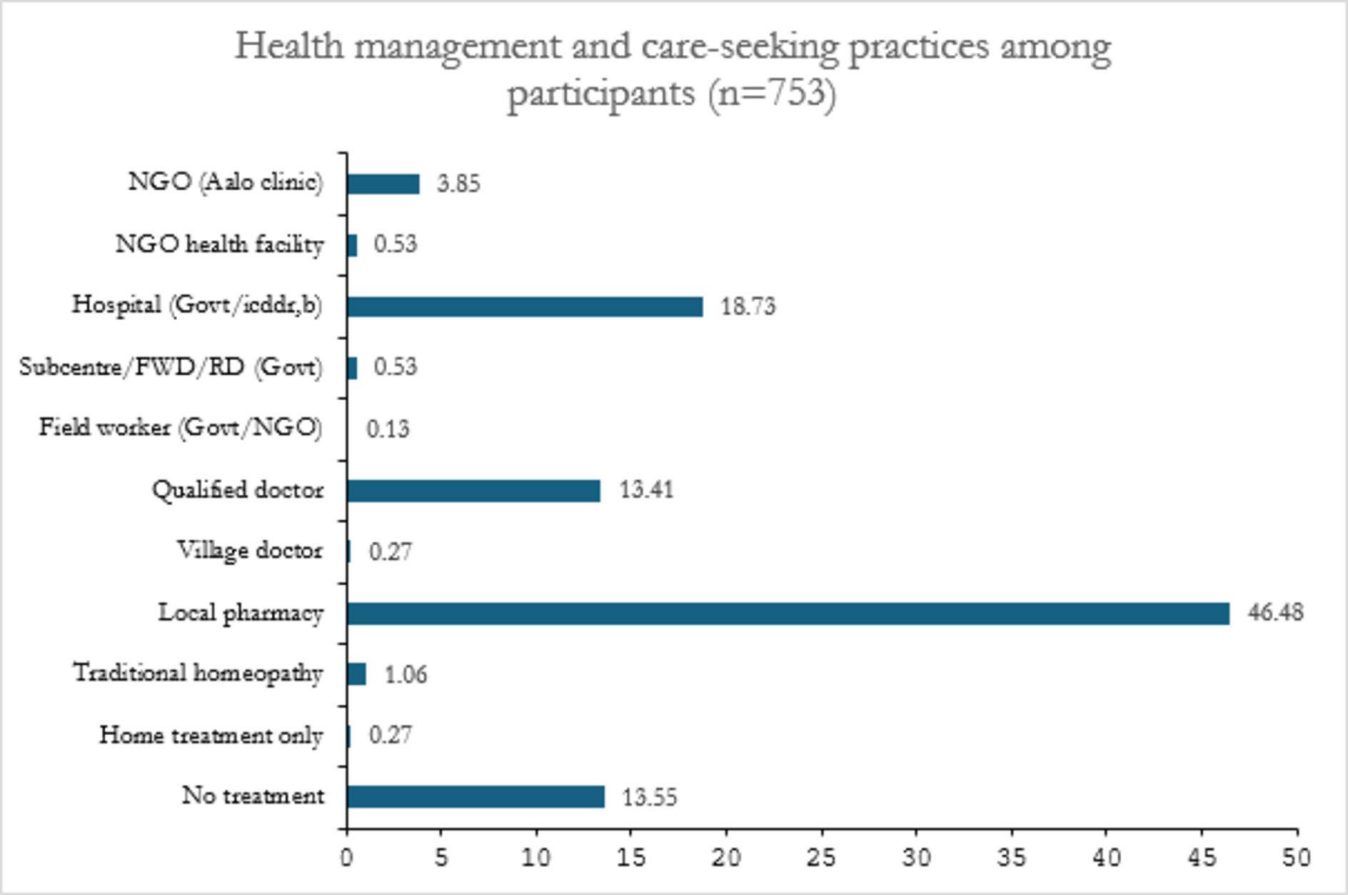
Percentage of Health Management and Care-Seeking Behavior for Children Under 2 in Urban Slums.

The majority of individuals sought medical care for their children from unqualified providers at local pharmacies (Fig. 3). A smaller proportion obtained treatment from hospitals (19%) or consulted with qualified doctors (13%). Notably, 14% of the children did not receive any form of treatment.

### Determinants of Care-seeking for ARI/Pneumonia-like symptoms

The following factors had a significant association with care-seeking from qualified doctors: age of the child, number of symptoms, duration of illness, maternal education, and household wealth (Table 2). Children aged 6–11 months had lower odds of receiving care (adjusted odds ratio (AOR) = 0.59, 95% CI 0.38–0.91) while those aged 12–23 months demonstrated a more pronounced decrease compared to those aged below six months. Children with multiple symptoms were twice as likely to receive care (AOR = 2.09, 95% CI 1.50–2.89), and those with illness lasting over seven days were 2.83 times (AOR: 2.83, 95% CI 1.59–5.05) more likely to receive care compared to those with fewer symptoms or shorter illness durations. Also, children of mothers with secondary or higher education were twice as likely to receive care (AOR: 2.07, 95% CI 1.12–3.82), and people belonging to higher wealth quintiles were significantly more likely to receive care from medically trained providers. Moreover, those living in a slum for 5–9 years (AOR = 1.81, 95% CI 1.03–3.18) were more likely to receive care than those living for less than five years.

**Table 2:** Factors associated with receiving care for ARI/pneumonia symptoms from qualified medical professionals (logistic regression: yes vs. no).

Variable	Unadjusted OR (95% CI)	Adjusted OR (95% CI)
Children’s characteristics		
Age of the child		
<6 months	Reference	Reference
6–11 months	0.65 (0.43–0.97)	0.58 (0.37–0.90)
12–23 months	0.41 (0.28–0.6)	0.36 (0.24–0.54)
Sex of the child		
Male	Reference	-
Female	0.96 (0.71–1.3)	-
Number of symptoms		
Single symptom	Reference	Reference
Multiple symptoms	2.32 (1.71–3.14)	2.09 (1.50–2.91)
Duration of illness		
<3 days	Reference	Reference
3–7 days	1.6 (0.93–2.73)	1.68 (0.96–2.96)
Above 7 days	2.61 (1.51–4.51)	2.83 (1.59–5.05)
Mothers’ characteristics		
Maternal age		
<25 years	Reference	Reference
25–34 years	1.39 (1.03–1.89)	1.42 (0.96–2.11)
35 years and above	1.07 (0.67-2.00)	1.13 (0.54–2.37)
Maternal education		
No formal education	Reference	Reference
Up to primary level	1.86 (1.06–3.26)	1.76 (0.96–3.23)
Secondary or higher level	2.29 (1.34–3.92)	2.07 (1.12–3.82)
Mothers’ working status		
Not working	Reference	Reference
Working	0.6 (0.37–0.97)	0.72 (0.42–1.22)
Household characteristics		
Number of household members		
Up to 4	Reference	Reference
5 or more	1.58 (1.16–2.16)	1.17 (0.74–1.83)
Household wealth quintile		
First (lowest)	Reference	Reference
Second (lower)	1.86 (1.02–3.39)	1.54 (0.82–2.91)
Third (middle)	2.9 (1.58–5.33)	2.31 (1.19–4.47)
Fourth (higher)	2.98 (1.73–5.13)	2.11 (1.15–3.88)
Fifth (highest)	4.27 (2.43–7.49)	2.80 (1.47–5.32)
Sex of the household head		
Male	Reference	-
Female	1.26 (0.8–1.96)	-
Age of the household head		
<30 years	Reference	Reference
30–39 years	1.41 (0.91–2.17)	0.89 (0.51–1.55)
40–49 years	1.91 (1.18–3.11)	1.13 (0.60–2.14)
50 years and above	1.62 (1.04–2.51)	0.77 (0.40–1.45)
Duration of living in the slum		
<5 years	Reference	Reference
5–9 years	1.80 (1.08–2.99)	1.81 (1.03–3.18)
10 or more years	1.68 (1.23–2.31)	1.30 (0.86–1.95)

*COR  * crude odds ratio. *AOR * adjusted Odds Ratio. *CI*Confidence Interval.

## Discussion

In this study, we investigated the determinants of care-seeking for childhood pneumonia among urban slum-dwellers in Bangladesh from qualified providers. We observed that most of the children did not receive care from qualified providers. Our findings illuminate several key determinants influencing care-seeking behavior, encompassing characteristics related to the child (i.e., age, number of symptoms, and illness duration), related to the mother (i.e., maternal education), and related to the household (i.e., wealth quintile). To our knowledge, this is the first epidemiological study to report a care-seeking pattern for childhood pneumonia among urban slum-dwellers in Bangladesh.

Our study reveals that children living in urban slums predominantly sought care from unqualified providers, like their rural counterparts^33–36^, However, in instances where the child’s illness was perceived as more severe, parents exhibited a diverse pattern of care-seeking, including qualified medical practitioners. This aligns with anthropological observations in South Asian countries, described as the simultaneous pattern of resort^37^. Our study further highlights that younger children with multiple symptoms and enduring illnesses were more likely to be taken to qualified providers. Intriguingly, we observed no statistically significant difference between male and female children in terms of care-seeking from qualified providers, unlike findings in rural studies, indicating a diminishing gender disparity in urban Bangladesh. A previous study conducted in a rural setting, however, found that seeking care for preterm newborns, whether from unqualified or qualified providers, was significantly lower for female children in comparison with male children^35^. In contrast, our study observed a diminishing gender disparity in care-seeking behavior among marginalized groups in urban slums. This suggests that different factors may be influencing gender disparities in these settings. Further research, particularly through qualitative studies, is needed to understand why this disparity exists in rural areas but not in urban slums. However, this may be attributed to an environmental effect originating from exposure to the behavior and mindset of educated urban people who treat their male and female children equally.

In contrast to the rural studies, maternal age did not emerge as a predictor of care-seeking for children from qualified providers in urban areas. Initial unadjusted analysis indicated that children whose mothers were employed were 40% less likely to receive care from qualified providers compared to those having non-working mothers. This discrepancy may be attributed to the challenges faced by working mothers in accessing qualified care providers, given their scarcity and distance from residence^21^. Additionally, the timing of public-sector health facilities may not be conducive to working mothers. However, this association lost statistical significance in the adjusted analysis. Conversely, a positive association between maternal education and care-seeking from qualified providers was observed, consistent with findings from multiple studies in Bangladesh and abroad^38,39^. Educated mothers having higher channels, increased mobility, and better access to information, exhibited a propensity to seek care from qualified providers for their children. Families with low wealth quintile face more barriers related to healthcare for children’s illnesses, including affordability, limited awareness, and inadequate infrastructure in Bangladesh^17,40,41^. Similarly, our study found the higher wealth quintile to be associated with a higher likelihood of seeking care. Ensuring a timely and appropriate diagnosis, along with sufficient antimicrobial treatment, is essential for the survival of critically ill patients with severe pneumonia^42^. Our study found that children who had pneumonia for seven days or fewer were less likely to seek medical treatment compared to those with a longer duration of illness of above seven days. Previous studies in Bangladesh have shown that pneumonia in children lasting more than seven days was associated with mortality^43^. Another study found that delayed treatment-seeking behavior, including seeking treatment ≥ 2 days after the onset of the disease, was more common in children who died from pneumonia^20^. The risk of death due to pneumonia, however, was higher in children with a long duration of illness (2–10 days) before death^26^.

Notably, in the urban Bangladeshi context of our study, factors such as age and gender of the household head, as well as the duration of stay in the slum, did not emerge as significant predictors of care-seeking from qualified providers. Initial unadjusted analysis suggested that children in larger households (with five or more members) were 58% more likely to receive care for pneumonia symptoms from qualified medical professionals than those in smaller households (having up to four members). This association, however, lost statistical significance in the adjusted analysis, indicating that household size alone is not a strong determinant of care-seeking behavior for children below two years of age with pneumonia in urban Bangladeshi slums. Therefore, focusing on other significant factors is crucial for developing effective strategies and interventions to improve access to qualified medical care. On the other hand, the consistent association between higher household income and increased care-seeking from qualified providers, as mirrored in various studies in Bangladesh and beyond, underscores the pervasive inequity within urban Bangladeshi slum-dwellers^22,44^.

### Strengths and limitations of the study

This study is based on a long-standing cohort study with several notable strengths. First, the sample size in the study was large, and it used standardized and validated sets of questionnaires. The participants were enrolled in a cohort study; therefore, we had a lack of uncertainty about the temporal association.

However, the limitations of the present study also warrant discussion. The data were based on self-reporting by mothers, which may have led to recall errors regarding the type of care used. There may also have been issues of social desirability bias, with mothers potentially providing answers they thought would please the interviewers. Although we accounted for a large number of study variables based on previously published reports and our data structure, we were unable to include a few more variables, including lack of the ability to pay, knowledge about the signs and symptoms of pneumonia, and availability of a qualified doctor in the area, because these variables were not parts of the data collected by the Urban HDSS.

### Significance of the study in policymaking and research

Further research, particularly through qualitative studies, is imperative to unravel the cultural and contextual factors contributing to the insufficient care-seeking behavior among urban slum residents from qualified providers. Given the positive association between maternal education and care-seeking from qualified providers, there is a pressing need to prioritize female education, considering its increasingly evident benefits. Simultaneously, health services should be tailored to accommodate the needs of working mothers, such as offering flexible working hours and establishing additional service outlets in closer proximity to urban poor settlements.

Crucially, while efforts to enhance child health are essential, achieving meaningful progress necessitates upstream policy initiatives that comprehensively address the structural determinants of health^45^ among the urban poor. To this end, additional health policy and systems research should be commissioned with a keen focus on evidence-based interventions. Implementation research should also be conducted to glean insights and replicate successful strategies, paving the way for scaling up these interventions across the entire country.

Moreover, the finding that just over half (53.92%) of those with ARI symptoms did not seek care from qualified providers, combined with the increased mortality risk associated with prolonged symptoms, reveals a significant gap in access to healthcare. This emphasizes the urgent need for targeted research and policy interventions to overcome barriers to timely access and seeking care from qualified providers and to improve health outcomes for the urban slum population.

## Conclusions

Our study sheds light on critical determinants influencing care-seeking behavior for childhood pneumonia among urban slum-dwellers in Bangladesh, revealing factors related to the child, the mother, and the household. Maternal education and higher income quintile emerged as positive predictors, emphasizing the need to prioritize female education and establish policies for ensuring equitable economic conditions. Our findings call for further research to unravel cultural and contextual factors affecting care-seeking behaviors among urban slum residents. Prioritizing female education and tailoring health services to meet the needs of working mothers, including flexible hours and additional service outlets, are crucial. Ultimately, to achieve meaningful progress in child health, comprehensive policy initiatives - such as improving access to healthcare, promoting maternal education, and addressing socioeconomic inequalities- are essential to address the structural determinants of health among the urban poor.

## Data Availability

The Urban HDSS data are not openly available but can be accessed through appropriate channels and by adhering to icddr, b’s data access policy, available at [www.icddrb.org]. For collaboration inquiries, please contact Abdur Razzaque at [razzaque@icddrb.org]. Additional information is accessible through the Urban HDSS website at [https://uhdss1.icddrb.org].

## Abbreviations

AOR: Adjusted odds ratio
AQP: Any qualified provider
ARI: Acute respiratory infection
BSCO: Bangladesh standard classification of occupations
CI: Confidence interval
FWC: Family welfare center
GAPPD: Global action plan for Pneumonia and diarrhoea
HDSS: Health and demographic surveillance system
HRH: Human resources for health
IMCI: Integrated management of childhood illness
ISCO: International standard classification of occupations
LMICs: Low- and middle-income countries
MOHFW: Ministry of health and family welfare
NGO: Non-governmental organization
NHS: National health service
OR: Odds ratios
RD: Rural dispensaries
SD: Standard deviation
SDGs: Sustainable development goals
UNICEF: United Nations children’s fund
WHO: World health organization
