# Correction: Determinants of care-seeking for ARI/Pneumonia-like symptoms among under-2 children in urban slums in and around Dhaka City, Bangladesh

**DOI:** 10.1038/s41598-025-30517-0

**Published:** 2025-12-22

**Authors:** Samiun Nazrin Bente Kamal Tune, Gulam Muhammed Al Kibria, Mohammad Zahirul Islam, Md. Arif Billah, Maya Vandenent, Md. Shamim Hayder Talukder, Ummay Ferhin Sultana, Maliha Khan Majlish, Shafiun Nahin Shimul, Margub Aref Jahangir, Jahangir A.M. Khan, Shahin Akter, Kazi Fayzus Salahin, Md. Razib Chowdhury, Abdur Razzaque, Taufique Joarder

**Affiliations:** 1 International Development Division (IDD), Abt Global, Dhaka, 1212 Bangladesh; 2 Johns Hopkins University Bloomberg School of Public Health, Baltimore, MD 21205 USA; 3 Development Cooperation Section, Embassy of Sweden, Dhaka, Bangladesh; 4 Health Systems and Population Studies Division, icddr,b, 68 Shaheed Tajuddin Ahmed Sarani, Mohakhali, 1212 Dhaka Bangladesh; 5 UNICEF, Dhaka, Bangladesh; 6 Eminence Associates for Social Development, Dhaka, Bangladesh; 7 Institute of Health Economics, University of Dhaka, Ramna, 1000 Dhaka Bangladesh; 8 Health Economics and Policy Unit, School of Public Health and Community Medicine, Institute of Medicine, University of Gothenburg, Gothenburg, Sweden; 9 SingHealth Duke-NUS Global Health Institute, Singapore, Singapore

Correction to: *Scientific Reports* 10.1038/s41598-024-80979-x, published online 29 March 2025

The original version of the Article contained errors.

The original version of this Article contained an error in the name of the author Samiun Nazrin Bente Kamal Tune, which was incorrectly given as Samiun Nazrin Kamal Tune.

Additionally, in the Author Information section,

“These authors contribute equally: Samiun Nazrin Bente Kamal Tune, Gulam Muhammed Al Kibria, Mohammad Zahirul Islam, Abdur Razzaque and Taufique Joarder”

now reads:

“Samiun Nazrin Bente Kamal Tune, Gulam Muhammed Al Kibria and Mohammad Zahirul Islam contributed equally to this work.

Abdur Razzaque and Taufique Joarder jointly supervised this work.”

The original Article has been corrected.

